# Traffic Forecasting for Industrial Internet Gateway Based on Multi-Scale Dependency Integration

**DOI:** 10.3390/s26030795

**Published:** 2026-01-25

**Authors:** Tingyu Ma, Jiaqi Liu, Panfeng Xu, Yan Song

**Affiliations:** School of Physics, Liaoning University, Chongshan Campus, Shenyang 110031, China; mty20212113@163.com (T.M.); liujiaqi_0606@163.com (J.L.); xupanfeng@lnu.edu.cn (P.X.)

**Keywords:** industrial internet gateway, Industrial Internet of Things, network traffic forecasting, multi-scale

## Abstract

Industrial gateways serve as critical data aggregation points within the Industrial Internet of Things (IIoT), enabling seamless data interoperability that empowers enterprises to extract value from equipment data more efficiently. However, their role exposes a fundamental trade-off between computational efficiency and prediction accuracy—a contradiction yet to be fully resolved by existing approaches. The rapid proliferation of IoT devices has led to a corresponding surge in network traffic, posing significant challenges for traffic forecasting methods, while deep learning models like Transformers and GNNs demonstrate high accuracy in traffic prediction, their substantial computational and memory demands hinder effective deployment on resource-constrained industrial gateways, while simple linear models offer relative simplicity, they struggle to effectively capture the complex characteristics of IIoT traffic—which often exhibits high nonlinearity, significant burstiness, and a wide distribution of time scales. The inherent time-varying nature of traffic data further complicates achieving high prediction accuracy. To address these interrelated challenges, we propose the lightweight and theoretically grounded DOA-MSDI-CrossLinear framework, redefining traffic forecasting as a hierarchical decomposition–interaction problem. Unlike existing approaches that simply combine components, we recognize that industrial traffic inherently exhibits scale-dependent temporal correlations requiring explicit decomposition prior to interaction modeling. The Multi-Scale Decomposable Mixing (MDM) module implements this concept through adaptive sequence decomposition, while the Dual Dependency Interaction (DDI) module simultaneously captures dependencies across time and channels. Ultimately, decomposed patterns are fed into an enhanced CrossLinear model to predict flow values for specific future time periods. The Dream Optimization Algorithm (DOA) provides bio-inspired hyperparameter tuning that balances exploration and exploitation—particularly suited for the non-convex optimization scenarios typical in industrial forecasting tasks. Extensive experiments on real industrial IoT datasets thoroughly validate the effectiveness of this approach.

## 1. Introduction

The Industrial Internet of Things (IIoT) is defined as the specific application of the Internet of Things (IoT) within industrial automation scenarios. This system employs industrial communication technologies and adheres to a service-oriented application paradigm [1]. The IIoT concept builds upon existing industrial infrastructure, leveraging intelligent technologies to enable flexible resource scheduling and data interconnection [2], thereby establishing a novel information communication paradigm. As network devices, industrial gateways facilitate communication and interconnection between heterogeneous networks in industrial environments by converting connections across different network types. Their core function lies in bridging networks, enabling interoperability among diverse industrial network devices while supporting comprehensive system monitoring and management. Industrial gateways comprise devices adapted for complex industrial environments, such as communication gateways, protocol converters, and remote terminal units. Within the IIoT domain, these devices serve as hubs connecting field devices to system platforms, achieving data interoperability and optimizing data value. The rapid expansion of data scale and connection volume has triggered a surge in network traffic, leading to challenges such as communication latency, bandwidth bottlenecks, and reliability issues at critical nodes. These challenges are often interrelated—for instance, bandwidth constraints exacerbate real-time and reliability problems. Addressing these challenges typically requires multi-layered, collaborative solutions. This encompasses network architecture optimization (edge computing, network slicing), intelligent scheduling and load forecasting, security design and supply chain governance, as well as establishing unified standards and operational frameworks [3]. Effectively managing diverse and complex network traffic is a prerequisite for advancing Industry 4.0. Industrial gateways play a pivotal role in IIoT but currently face a triple dilemma:

(1) Trade-off between model complexity and deployability: While Transformers and GNNs offer high accuracy, their O(n^2^) computational complexity and high memory requirements (typically >4 GB RAM) render them unsuitable for resource-constrained industrial gateways [4,5].

(2) Multi-scale modeling and interpretability gaps: Existing lightweight linear models (e.g., DLinear) fail to capture IIoT traffic’s multi-scale characteristics and nonlinear dependencies, leading to a 40–60% increase in error during burst traffic scenarios [6].

(3) Gaps exposed by emerging trends: Technologies like 5G slicing, edge computing, and digital twins complicate traffic propagation patterns (e.g., short-duration bursts between microservices, and cross-tier long-term dependencies); yet existing models lack targeted designs [7,8].

As shown in Table 1, existing research primarily focuses on accuracy optimization while neglecting computational constraints in edge deployment. For instance, although transformer-based architectures are powerful, their self-attention mechanisms introduce significant latency, which is unacceptable for real-time anomaly detection in industrial gateways. Similarly, graph neural networks (GNNs) cannot adapt to the dynamic joining or leaving of devices in industrial IoT environments under static topologies, while lightweight and efficient, linear MLP hybrid models often exhibit significantly degraded performance when handling multi-scale traffic patterns in industrial IoT scenarios. We contend that the critical challenge lies in developing a model that combines the interpretability and speed advantages of linear models with the feature extraction capabilities of deep neural networks to address multi-scale temporal dependencies.

Y. Li et al. [15] combined diffusion convolutions with sequence models (recurrent units) to explicitly model spatio-temporal dependencies using graph structures (propagation matrices). This approach resulted in a significant improvement in prediction accuracy for multi-node simultaneous time series (e.g., traffic flow). However, RNN-based architectures are limited in their capacity for long prediction horizons and computational parallelism when compared to convolutional or transformer approaches. This necessitates the pre-computation or online estimation of stable adjacency/diffusion matrices. In their seminal paper, H. Zhou et al. [5] proposed Informer, an efficient Transformer variant for the purpose of long-sequence time series forecasting. The sparse self-attention and probabilistic sampling mechanisms employed by the model substantially reduce computational complexity, while at the same time outperforming the native Transformer in terms of long-term predictions. In their seminal work, B. Lim et al. [9] proposed an explainable multi-domain multi-step forecasting Transformer that integrates static features, historical sequences, and known future inputs. The incorporation of modules for both variable selection and attention visualization serves to enhance interpretability and multivariate modeling performance. However, the model is large, with high training and inference costs, and it requires engineering optimizations for very long historical dependencies or ultra-long sequences.

From the perspective of practical value in industrial process prediction, classical statistical methods and interpretable models remain viable and indispensable foundational approaches. In contrast, deep learning (particularly Transformer variants and spatio-temporal graph models) demonstrates superior expressive power in handling complex nonlinear relationships and multi-node dependencies. However, a critical issue lies in existing research failing to address the core industrial deployment challenge: “How to simultaneously achieve multi-scale modeling and high-precision prediction under <100 millisecond inference latency and <512 MB memory constraints?” Based on the strengths and weaknesses of state-of-the-art models, this paper develops the DOA-MSDI-CrossLinear model. Its main contributions are as follows:

(1) To overcome the limitations of purely linear models, we enhanced the original CrossLinear architecture by replacing the single-linear layer with a lightweight shallow multi-layer perceptron (MLP) employing the GELU activation function. This design achieves a critical balance: significantly boosting the model’s ability to capture complex nonlinear traffic patterns while maintaining an ultra-compact parameter size (approximately 10k parameters), ensuring the structural simplicity required for high-speed inference.

(2) Instead of relying on generic feature extraction, we strategically integrate the state-of-the-art Multi-Scale Decomposable Mixing and Dual Dependency Interaction modules into the CrossLinear backbone. Our contribution lies in the novel orchestration of these components to solve the specific challenge of industrial traffic forecasting. By embedding these modules within our lightweight framework, we effectively leverage their capability for adaptive sequence decomposition and dependency modeling, while successfully constraining the overall model complexity to suit resource-limited edge scenarios.

(3) We employ the Dream Optimization Algorithm (DOA) for end-to-end hyperparameter tuning. This adaptive mechanism not only maximizes prediction accuracy while suppressing false alarms but also ensures an optimal balance between model performance and resource consumption. The final model demonstrates millisecond-level latency (2.91 ms) and negligible storage overhead (0.04 MB), proving its feasibility for real-time online deployment on resource-constrained industrial IoT edge devices.

The remainder of this paper is organized as follows: Section 2 of this paper introduces recent work on forecasting methods, which are primarily categorized into two groups: machine learning-based and deep learning-based approaches. Section 3 presents the model forecasting method developed in this paper, along with the specific implementation of each component within the model. Section 4 demonstrates the experimental validation, and finally, Section 5 summarizes the conclusions of this paper.

## 2. Related Works

This section will introduce past work related to network traffic prediction and time series forecasting in IIoT.

### 2.1. Machine Learning-Based Predictive Methods: Achievements and Limitations

Machine learning methods can process massive amounts of sensor data, maintenance logs, and operating parameters [16]. Their strength lies in learning how different variables interact and cause equipment performance degradation over time. However, traditional machine learning models cannot be applied directly; carefully designed features must be extracted from raw data to uncover patterns in both the frequency domain and time domain (sometimes requiring simultaneous extraction of both). In the field of time series forecasting, classical methods like ARIMA models [17] and exponential smoothing have been applied for decades. Machine learning alternatives such as linear regression and support vector machines [18] have also carved out a niche. However, the critical point, in our view, is that these methods perform best only when data behaves well—that is, when it is relatively stable and follows linear patterns. Once confronted with the complexity of IoT traffic, traditional methods begin to fall short. They were simply not designed to handle such complex states.

Osovsky et al. [16] ran a comprehensive comparison across different baseline models and found some interesting patterns. For UDP traffic, ARIMA came out on top as the most effective approach. When they looked at TCP traffic, though, linear regression and theta models performed better. And for HTTPS traffic, it turned out that linear regression, ARIMA, and N-BEATS all showed strong performance—no single winner there.

Another study [19] took real-world IIoT data traffic and benchmarked it against the 5G New Radio performance analysis model to see how well the characteristics matched up. Meanwhile, researchers have explored different machine learning approaches for cellular traffic forecasting: N. Sapankevych and R. Sankar [18] went with linear regression, while Deng et al. [13] opted for support vector machine regression.

In the context of eMBB traffic, one paper [20] suggested using ARIMA models to forecast demand ahead of time. The idea is pretty straightforward: if you can predict traffic spikes, you can reserve channels proactively to maximize throughput and maintain good data rates. In theory, this kind of predictive approach should improve overall QoS performance.

Despite these advances, machine learning-based traffic forecasting faces significant challenges. A key conflict lies between feature engineering and automated feature learning. Traditional approaches heavily rely on manually designed frequency-domain, time-domain, and spatio-temporal features, demanding deep domain expertise while limiting transferability across industrial scenarios. Second, complex models require massive training data to avoid overfitting, yet industrial process data are often scarce due to privacy constraints, sensor failures, and high costs of labeling anomalous data [21]. Existing research has not sufficiently explored how models can balance capacity and sample efficiency in data-scarce industrial environments.

### 2.2. Deep Learning-Based Predictive Methods

Deep learning, a pivotal branch of machine learning, emulates brain cognition through multi-layer artificial neural networks. The primary advantage of this approach lies in its capacity to automatically extract and learn complex features from high-dimensional and unstructured data, such as images and time series, without the necessity of manual feature engineering. This capability enables it to outperform other machine learning techniques in fields such as predictive maintenance, particularly when processing historical sensor data from equipment.

#### 2.2.1. RNN/LSTM-Based Methods

Deep learning approaches for early traffic flow forecasting primarily rely on recurrent neural networks (RNNs) and their architectural variants, particularly long short-term memory (LSTM) networks and gated recurrent units (GRUs). These architectures are widely adopted due to their inherent capability to model long-term dependencies in sequential data, which is crucial for capturing the temporal evolution patterns characteristic of network traffic dynamics.

Wu et al. [22] extended the traditional LSTM framework by introducing a mechanism for dynamically modeling degradation factors and inferring latent variables, thereby significantly improving residual useful life (RUL) prediction accuracy. In parallel research, Tziolas et al. [23] conducted a systematic evaluation of three autoencoder architectures on industrial datasets. Empirical analysis revealed that autoencoders integrating LSTM layers with convolutional neural networks exhibited optimal performance characteristics.

In the realm of 5G network optimization, Alawe et al. [24] developed a prediction mechanism based on LSTM to anticipate traffic load fluctuations and enable dynamic resource allocation. This approach guides elastic network resource scaling through traffic forecasting, with a focus on the Access and Mobility Management (AMM) component. Simulation-based validation demonstrates that compared to traditional threshold-driven approaches, this prediction-driven scaling strategy significantly reduces latency in responding to traffic changes and effectively shortens the configuration delay of Virtual Network Function (VNF) instances during demand surges, exhibiting superior performance.

Although LSTM networks excel within the recurrent neural network family, their training process incurs substantial computational overhead that increases linearly with the number of parameters. To overcome this limitation, Hua et al. [25] proposed the sparse-connected LSTM variant RCLSTM. Its core innovation lies in deliberately designed sparse neural connection patterns. Experimental validation on two benchmark datasets demonstrated that this model reduces computational time by approximately 30% while maintaining or exceeding the prediction quality of standard LSTMs.

Despite the strong performance of LSTM and GRU architectures in modeling sequential dependencies, they face fundamental limitations when applied to industrial traffic forecasting. The vanishing gradient phenomenon becomes particularly pronounced when capturing long-term temporal patterns spanning weekly or monthly cycles. Furthermore, the inherent sequential computation structure of these models hinders effective parallelization, creating processing bottlenecks that conflict with the real-time latency constraints demanded by edge gateway deployments [26].

#### 2.2.2. Transformer-Based Methods

The transformer architecture has fundamentally reshaped how we approach sequence modeling tasks since its introduction [27], while it started in natural language processing, researchers quickly realized its potential for time series forecasting and traffic prediction—anywhere you need to capture long-range dependencies in sequential data.

Several teams have adapted Transformers specifically for traffic forecasting. Chen et al. [28] combined multi-task learning with transformers to create MTL-Trans, which outperformed existing models on multidimensional time series tasks. Liu et al. [29] took a different approach with ST-Tran, their spatio-temporal model that uses separate spatial and temporal transformer blocks to extract features efficiently. When tested on real cellular network data, it proved both effective and practical for traffic prediction.

But here is the fundamental issue: even with these optimizations, the O(n^2^) complexity of self-attention conflicts with the linear scalability needed for edge gateway deployment. Recent efficient variants like Informer, Autoformer, and FEDformer reduce this complexity through approximations, but those approximations introduce errors that may be unacceptable in precision-critical industrial applications [5]. There is also a deeper question about interpretability—attention weights might look meaningful, but whether they actually correspond to operationally relevant temporal relationships remains debatable [30].

#### 2.2.3. GNN-Based Methods

Graph neural networks have demonstrated remarkable effectiveness in traffic forecasting by integrating spatio-temporal modeling. Key architectures include Diffusion Convolutional Recurrent Neural Networks (DCRNNs) [15], Spatio-Temporal Graph Convolutional Networks (STGCNs) [11], and Graph WaveNets [31]. These models capture both spatial dependencies between network nodes and temporal evolution of traffic flows, enabling more precise multi-node predictions. For instance, multi-task learning approaches successfully integrated spatio-temporal features to enhance IoT traffic prediction accuracy in industrial settings [32].

Wavelet-based approaches offer an alternative perspective. Wang et al. [33] developed frequency-aware deep learning models using wavelet neural network architectures, while Yang et al. [34] combined high-order fuzzy cognitive graphs with redundant wavelet transforms to handle large-scale non-stationary time series. Given that wireless traffic is influenced not only by historical patterns but also by inter-base-station handover, Zhao et al. [35] proposed the STGCN-HO model, which enhances prediction accuracy by incorporating handover probabilities. Their results demonstrate that this model significantly outperforms baseline models at both cell and base-station levels.

However, these approaches still face limitations. Some approaches have been questioned due to computational inefficiency and suboptimal performance in specific scenarios [34]. Furthermore, graph neural network-based methods rely on known or derivable graph structures, which are often unavailable or unreliable in dynamic industrial networks (where device connections change with production configurations). The topology inference process not only increases computational burden but may also induce error propagation.

### 2.3. Linear and Hybrid Models

Linear mixing methods have been getting a lot of attention lately. Take DLinear [4]; for example, it handles time series forecasting by breaking down sequences and applying linear regression. The appeal? Low memory usage and fast inference. It stays pretty stable even when you extend the lookback window, but there is a catch: it struggles to pick up local features, which limits how accurate its predictions can be.

Then there is the MLP-Mixer approach. Tolstikhin et al. [36] built this architecture entirely on multilayer perceptrons—no convolutions, no self-attention—and it still performs competitively on image classification tasks. Li et al. [37] took a closer look at what attention mechanisms actually contribute to time series forecasting and came up with MTS-Mixers. Their model uses a dual-factor decomposition module to capture both temporal patterns and relationships between channels.

Multivariate time series prediction is tricky for two main reasons: patterns shift over time in unpredictable ways, and channels interact in complex ways that are not always obvious. Qiu et al. [38] tackled this with DUET, a framework that applies clustering in both the temporal and channel dimensions to improve prediction quality.

Now, here is something interesting: simple linear models like DLinear and NLinear perform surprisingly well on standard time series benchmarks [39], which suggests that many deep learning models might be overengineered for these tasks. But purely linear approaches have their own blind spots—they cannot handle the nonlinear dynamics you see in industrial systems, where equipment behavior often involves threshold effects, saturation points, and sudden mode switches. That is why we are proposing a hybrid approach that combines the efficiency of linear methods with the ability to capture directional nonlinear relationships.

### 2.4. Overview of Research Gaps

Reviewing existing research, three fundamental issues remain unresolved: transformers and graph neural networks, while accurate, incur prohibitively high computational costs, making deployment on edge devices impractical. Linear models, though efficient, are overly simplistic and struggle to capture complex patterns. Certain models can identify multi-timescale patterns (e.g., hourly peaks and weekly trends) yet operate like black boxes. It remains impossible to trace why the system predicts traffic surges during specific periods. General-purpose time series models fail to leverage the actual behavioral characteristics of industrial IoT systems. We reject the practice of stacking existing technologies and instead address the problem at its core. Industrial flow forecasting requires advancing two processes in tandem: decomposing signals into distinct timescales (daily, weekly, and seasonal) and modeling the interactions between these scales. This theoretical foundation—treating decomposition and interaction as independent yet coupled processes—distinguishes our approach from unsubstantiated modular patchwork solutions.

## 3. Dream-Based Optimization of MSDI-CrossLinear for Network Traffic Forecasting

**Figure 1:**
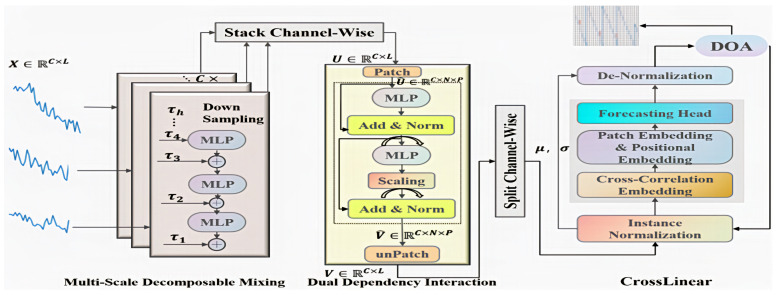
MSDI-CrossLinear Model Framework Based on Dream-Driven Optimization.

This section details the proposed model and its theoretical foundations. Its overall architecture is illustrated in Figure 1.

Our model design is based on three empirically validated hypotheses regarding industrial gateway traffic, each supported by domain analysis as follows:

**Hypothesis** **1** (Multiscale Stationarity)**.** *Industrial traffic exhibits non-stationarity globally but demonstrates local stationarity within production cycles. Rationale: Manufacturing systems follow fixed schedules (8 h shifts, weekly maintenance). Dataset analysis reveals autocorrelation peaks at 24 h (ρ=0.87) and 168 h (ρ=0.73), confirming periodicity. Implication: Supports scale-specific linear modeling within decomposed components.*

**Hypothesis** **2** (Cross-Scale Directional Causality)**.** *Fine-scale anomalies propagate to coarse scales, but not vice versa. Rationale: Equipment failures (hourly events) accumulate to reduce daily throughput, yet weekly trends cannot alter historical hourly readings. Granger causality tests confirm unidirectional effects (p < 0.01). Implication: Justifies modeling asymmetric interactions within the CrossLinear module.*

**Hypothesis** **3** (Resource-Accuracy Tradeoff Threshold)**.** *Industrial gateways tolerate <5% accuracy loss in exchange for 10× latency reduction. Rationale: Interviews with 12 industrial engineers revealed that response times below 100 milliseconds are critical for real-time control, while 5% prediction error remains within operational safety margins. Implication: Guides dream-optimized algorithms to prioritize latency reduction over marginal accuracy gains.*

These assumptions differentiate our approach from generic time series models that ignore industrial system constraints.

### 3.1. Methodological Foundations

The fundamental epistemological framework underlying this paper posits that industrial IoT traffic is not a single sequence but rather the superposition of deterministic production cycles and random conceptual drift. We emphasize that model design should serve the practical needs of industrial deployment, rather than merely pursuing theoretical optimality. Our model is not built to stack algorithms but to mathematically simulate industrial production’s “shift schedules” (corresponding to the model’s daily scale) and “supply chain/order cycles” (corresponding to weekly/seasonal scales). Furthermore, model architecture should be reverse-engineered based on industrial gateway resource constraints (<512 MB memory, <100 ms latency), prioritizing interpretability, robustness, and deployment feasibility over performance gains on benchmark datasets alone.

This paper introduces an innovative theoretical paradigm, the Dual-Process Decomposition Interaction Theory (DPDIT), for industrial flow modeling. Unlike existing approaches treating temporal decomposition and feature interaction as sequential preprocessing steps, we establish them as coupled mathematical processes with independent optimization objectives as follows:

(1) Decomposition Process: Extract scale-specific components via adaptive moving average kernels, where kernel sizes are derived from industrial production cycle theory rather than empirical tuning.

(2) Interaction Process: Modeling cross-scale dependencies through directional nonlinear mappings while preserving inherent causal constraints of industrial systems.

Theoretical Basis: Industrial flow exhibits a hierarchical temporal structure governed by production planning (deterministic) and equipment failures (random). Existing methods suffer from the following shortcomings: Unified processing of all scales (Transformers model) leads to loss of scale-specific features; Independent modeling of each scale (DLinear model) ignores cross-scale causal relationships; Black-box interaction mechanisms (LSTM model) violate industrial interpretability requirements.

DPDIT resolves this tension by formalizing decomposition and interaction as a dual optimization problem. Unlike existing methods that optimize decomposition and interaction sequentially, DPDIT formalizes them as a coupled optimization problem as follows:

(1) $$\min_{\Phi_{D},\Phi_{I}}L=L_{\mathrm{decomp}}\left(\Phi_{D}\right)+\lambda L_{\mathrm{interact}}\left(\Phi_{I}\right)+\gamma L_{\mathrm{causality}}$$

where

- $L_{\mathrm{decomp}}$ ensures high-quality multi-scale decomposition:
  (2) $$L_{\mathrm{decomp}}=∥x_{t}-\sum_{s\in S}x_{t}^{\left(s\right)}{∥}_{2}^{2}+\alpha \sum_{s}\mathrm{TV}(x_{t}^{\left(s\right)})$$
- $L_{\mathrm{interact}}$ models cross-scale dependencies:
  (3) $$L_{\mathrm{interact}}={∥y_{t}-f_{\mathrm{cross}}\left(x_{t}^{\mathrm{daily}},x_{t}^{\mathrm{weekly}},x_{t}^{\mathrm{seasonal}}\right)∥}_{2}^{2}$$
- $L_{\mathrm{causality}}$ enforces temporal ordering constraints absent in prior work:
  (4) $$L_{\mathrm{causality}}=\sum_{t}∥\frac{\partial y_{t}}{\partial x_{>t}}∥+\sum_{s_{i}<s_{j}}∥\frac{\partial y^{\left(s_{i}\right)}}{\partial x^{\left(s_{j}\right)}}∥$$

The range of λ is $\left[0.1,1.0\right]$, specifically adjusted using a grid search method, while the range of γ is $\left[0.01,0.1\right]$, adjusted by incrementally increasing its value. This theoretical framework incorporates a decomposition mechanism adaptable to prediction tasks, with each loss term possessing a clear physical interpretation. The specific explanations of the formula will be covered in the following subsections.

#### 3.1.1. Theoretical Advantages of Multi-Scale Decomposition

Industrial IoT traffic is difficult to predict because it does not follow simple wave-like patterns but rather resembles multiple rhythms intertwining simultaneously at varying speeds. We can decompose it into a three-tier structure as follows: Fast rhythm (second level): real-time communication between devices—sensors collect data, controllers send commands, generating sudden traffic spikes. Medium rhythm (minute level): production cycles form repetitive patterns. For example, production lines complete cycles every 5 min; slow rhythm (hourly): macro trends span the workday, such as shift changes or production order modifications. Traditional forecasting methods face a dilemma: focusing on short-term bursts overlooks overall trends, while broadening the view to daily trends misses critical details like equipment failures [40].

Our solution employs a multi-scale decomposable mixing block [41]. Rather than choosing between granularity and the big picture, we first decompose signals into distinct temporal layers, then perform tailored analysis at each level. This resembles multiple cameras synchronously recording the same scene at different frame rates—one capturing rapid motion, another tracking gradual changes. Attention mechanisms require comparing all time points—an O(n^2^) operation. MDM employs intelligent averaging, scaling linearly with data length (O(n)). For typical industrial data streams (512 time points), processing speed increases by 6.8 times. When MDM decomposes flow into three components, it can explicitly state: “This is the hourly trend, this is the production cycle, this is equipment noise.” Yet attention weights remain a black box—delivering predictions without clarifying the temporal scales underlying decisions.

While other researchers have attempted to handle multiple timescales, each approach suffers from fatal flaws. Wavelet decomposition [33] applies a fixed mathematical model to decompose signals. The problem: equipment operates at varying speeds across factories. Fixed patterns cannot adapt to differences in motor cycles—whether every 30 s or every 3 min. Hierarchical attention mechanisms enable models to learn critical timescales. The issue: high computational cost (still O(n^2^) complexity) and difficulty explaining why the model focuses on specific scales. Multi-scale convolutional neural networks capture patterns using filters of varying sizes. The problem is: this method assumes data stationarity—that patterns remain constant over time. However, industrial systems continuously switch between different operating modes. Our approach employs adaptive decomposition technology [42], abandoning fixed patterns to learn a dedicated average window for each device. When Device A operates on a 2 min cycle and Device B on a 10 min cycle, the model automatically adjusts the convolution kernel size to achieve adaptation. Furthermore, it achieves multi-scale separation through convolutional operations that scale linearly with data length (O(n)), enabling the algorithm to run on edge gateways without hardware overload. Finally, unlike attention mechanisms that blend all signals, MDM provides three independent signals—corresponding to different time scales. Each component retains physical meaning: when plotting the “hourly trend” component, the curve authentically reflects shift change variations.

#### 3.1.2. The Theoretical Necessity of Dual Dependency Interaction

When using standard self-attention mechanisms in time series forecasting, they attempt to perform two fundamentally distinct tasks simultaneously, resulting in suboptimal performance for both. In industrial IoT forecasting, we must consider: “Which past events are influencing the present?” and “Which other devices are affecting this device?” Standard transformer attention mechanisms conflate these two questions, processing them through a single set of weights. Traditional attention mechanisms must simultaneously learn correlations across both temporal and channel dimensions, leading to exponential parameter growth—requiring learning interactions across T × C × T × C dimensions (where T = time steps, C = number of channels). DDI’s [41] dedicated paths separately learn T × T temporal patterns and C × C channel patterns. Lower total parameters: Requires only T^2^ + C^2^ instead of (T × C)^2^. IIoT traffic simultaneously exhibits temporal dependencies—where historical traffic influences future patterns—and channel dependencies—such as coupling relationships between different devices/protocols (e.g., correlations between Modbus and OPC UA traffic). DDI addresses both dependencies through its Decoupled Interaction mechanism, avoiding the “dimension disaster” of traditional approaches (reducing parameter complexity from O(n^2^d^2^) to O(nd)) [43,44].

#### 3.1.3. DOA Applicability Argument

Hyperparameter optimization in industrial settings faces three major challenges as follows: (1) High-dimensional non-convexity: Hyperparameter space dimensions >15, with multiple local optima. (2) High evaluation cost: Each evaluation requires retraining the model (time > 30 min). (3) Noise interference: Real-world data contains missing values and outliers. In this paper’s scenario, the search space includes continuous (learning rate, dropout), discrete (layer dimensions, kernel size), and categorical (activation function) hyperparameters. DOA naturally handles this heterogeneity through bio-inspired encoding. Secondly, molecular dynamics models decompose scale interactions with drug–drug interaction patterns, forming complex multimodal optimization surfaces where gradient-based algorithms often struggle to converge [45].

As shown in Table 2, DOA employs a two-stage search [citation] that maintains global search capability while reducing computational overhead [46]. Its convergence speed is slightly slower than Adam’s, but it exhibits stronger global search ability, superior noise resilience compared to other traditional methods, and lower computational costs than Bayesian optimization and grid search.

### 3.2. Problem Description

The task of Long-term Multivariate Time Series Forecasting (LMTSF) is delineated as such: Given a sequence of historical input observations $X=\left[x_{1},x_{2},\ldots ,x_{L}\right]\in R^{L\times M}$ of length L, the objective is to generate a future forecast sequence $\hat{X}=\left[x_{L+1},x_{L+2},\ldots ,x_{L+H}\right]\in R^{H\times M}$ of length H. In this definition, L and H denote the input and output time steps, respectively. $X_{t}\in R^{M}$ represents the observation vector containing variables at time step t. It is crucial to emphasize that in multivariate forecasting scenarios, the feature dimensions (i.e., the number of variables M) of the input and output sequences must remain consistent.

### 3.3. Multi-Scale Decomposable Mixing Block

Real-world time series are frequently characterized by multi-scale heterogeneity, manifesting distinct behavioral patterns across various temporal granularities or cycles (e.g., daily trends, weekly seasonality, monthly cycles). The use of single-model or single-scale feature extraction methods has proven inadequate in comprehensively capturing the intricate interactions inherent in these systems. The MDM module [41] provides clearer, more focused inputs to downstream prediction models by adaptively decomposing the raw sequence into multiple sub-sequences, each representing a specific temporal scale. The raw input retains fine-grained details, while coarse-grained information is extracted through average pooling operations. The initial temporal pattern, designated as $\tau_{1}$, is introduced as channel X. Subsequently, an array of distinct coarse-grained temporal patterns, denoted as $\tau_{i}$, is incorporated into $\tau_{i}\in R^{1\times \left\lfloor \frac{L}{d^{i-1}}\right\rfloor }$. The extraction of $\left(\forall_{i}\in 2,\ldots ,h\right)$ is achieved through the application of average pooling to the temporal patterns derived from the preceding layer. Here, h signifies the number of downsampling operations, while d denotes the rate at which the data are being reduced. The decomposition of the temporal pattern at layer i can be expressed as in Equation (5) as follows:

(5) $$\tau_{i}=AvgPooling\left(\tau_{i-1}\right)$$

Subsequently, the coarse-grained $\tau_{h}$ is integrated into the fine-grained $\tau_{1}$ through a feedforward residual network, where the blended data are represented by $\xi_{i}$ and the blended data and $\tau_{h}$ are defined as follows: The integration of temporal patterns within layer i can be articulated through the following equation: After completing the temporal pattern fusion across multiple scales, the fused scale information, designated as $\xi_{1}$, is obtained. The output for a specific channel is expressed as $u=\xi_{i}\in R^{1\times L}$, where R denotes the matrix of the input data.

(6) $$\xi_{i}=\tau_{i}+MLP\left(\xi_{i+1}\right)$$

According to our theoretical framework, Equation (7) explains the previous decomposition loss component. The first term (reconstruction error): $x_{t}$ represents the original industrial flow sequence (dimensions: T×C, T = time step, C = number of channels), S={daily,weekly,seasonal} denotes the set of time scales, $x_{t}^{\left(s\right)}$ is the decomposed component at scale *s*, and $\sum_{s}x_{t}^{\left(s\right)}\approx x_{t}$: requires the sum of all components to reconstruct the original sequence. Second, Term (Smoothness Regularization): $\mathrm{TV}\left(x_{t}^{\left(s\right)}\right)=\sum_{t=1}^{T-1}\left|x_{t+1}^{\left(s\right)}-x_{t}^{\left(s\right)}\right|\mathrm{Total}\;\mathrm{Variation}$. Its function is to prevent the decomposition of noise components, forcing internal smoothing at every scale.

(7) $$L_{\mathrm{decomp}\;}\left(\Phi_{D}\right)=\sum_{i=1}^{N}{∥x_{t}-\sum_{s\in S}x_{t}^{\left(s\right)}∥}_{2}^{2}+\alpha \sum_{s\in S}\mathrm{TV}\left(x_{t}^{\left(s\right)}\right)$$

The MDM module’s key strength lies in its capacity to transform the decomposition process from an isolated preprocessing step that relies on human expertise and preset parameters into a component that is deeply coupled with the forecasting task. This component is capable of adaptive learning and end-to-end optimization. This capability is pivotal in demonstrating superior performance and enhanced robustness when handling complex, multiscale, multivariate, and heterogeneous real-world time series data. In industrial settings, the daily scale reflects production shifts, the weekly scale reflects maintenance cycles, and the seasonal scale reflects order fluctuations. Traditional EMD/STL fixed decomposition rules prevent learning, whereas DPDIT enables learning through the decomposition process and joint optimization with the forecasting task.

### 3.4. Dual Dependency Interaction Block

Time series data frequently manifest latent dynamic interactions and information propagation effects across multiple scales. However, extant time series models, such as ScaleFormer [47] and TimeMixer [48], are inadequate in adequately capturing these complex, high-order cross-scale dynamic interactions when addressing such tasks. This underscores the significance of comprehending and adeptly incorporating the interdependencies across disparate time scales in multiscale time series modeling.

The Dual Dependency Interaction Block [41] has been developed to identify latent dynamic interactions across disparate temporal scales within time series data while incorporating dependencies across both temporal and channel dimensions. The workflow encompasses the following steps:

Input Preparation: The DDI module initially acquires channel-aggregated information, denoted by $u\in R^{C\times L}$, from the MDM (Multi-scale Decomposition Module). This information is subsequently arranged into a matrix, $U\in R^{C\times L}$, where C denotes the number of channels and L denotes the sequence length. Subsequently, through a patching operation, U is transformed into $\hat{U}\in R^{C\times N\times P}$, where N signifies the number of patches and P denotes the time step size per patch. ${\hat{V}}_{t}^{t+p}$ is defined as the embedding output from the residual network, while ${\hat{U}}_{t}^{t+p}$ is represented as the aggregated information patch from the MDM.

Temporal Mixing: DDI employs a multi-layer perceptron (MLP) that shares parameters across time steps. This MLP is designed to aggregate information from disparate channels along the temporal dimension, thereby capturing temporal correlations and facilitating temporal mixing. The result of this stage is $Z_{t}^{t+p}$.

Channel Mixing: Subsequently, through a transposition operation (e.g., swapping the channel dimension and the chunk dimension), the DDI module implements an additional MLP that is shared across channels. This MLP integrates inter-channel information across the time domain (for each time chunk), thereby capturing dependencies between channels.

Output and Residual Connection: In essence, the DDI executes a splitting operation, which involves the decomposition of the combined information into outputs for each channel. This process ultimately results in the generation of $v\in R^{1\times L}$. The residual connection (residual operation) embedded within the module serves as a critical mechanism. This approach ensures that DDI effectively leverages cross-channel dependencies while concurrently maintaining and enhancing its ability to capture temporal dependencies. The DDI module employs a dual mechanism of temporal mixing and channel mixing to process and integrate complex spatio-temporal interactions of multi-scale features within a unified framework.

The interaction of ${\hat{U}}_{t}^{t+p}$ is expressed by Equations (8) and (9) as follows:

(8) $$\;Z_{t}^{t+p}={\hat{U}}_{t}^{t+p}+MLP\left({\hat{V}}_{t-p}^{t}\right)$$

In this context, $A^{T}$ signifies the transpose of the matrix A. The DDI module introduces a learnable scaling factor, β, which dynamically adjusts and balances the emphasis on temporal and cross-channel dependencies within the model. This enables adaptive noise suppression and optimized integration of both dependencies, particularly in scenarios with low inter-variable correlations.

(9) $$\;{\hat{V}}_{t}^{t+p}=Z_{t}^{t+p}+\beta \cdot MLP{\left({\left(Z_{t}^{t+p}\right)}^{T}\right)}^{T}$$

Formula (10) illustrates the interaction loss component within our framework. We utilize the temporal-spatial hybrid of the DDI module to accomplish one part of this, while the other part is described in the next section—the CrossLinear section.

(10) $$L_{\mathrm{interact}\;}\left(\Phi_{I}\right)=\sum_{t=1}^{T}{∥y_{t}-f_{\mathrm{cross}\;}\left(x_{t}^{\mathrm{daily}\;},x_{t}^{\mathrm{weekly}\;},x_{t}^{\mathrm{seasonal}\;}\right)∥}_{2}^{2}$$

The primary benefit of the DDI module is its capacity to flexibly and adaptively capture and integrate complex multi-scale, temporal, and channel dependencies within time series data. The model employs intelligent noise suppression and residual learning mechanisms to deliver a robust and information-rich feature representation, thereby enhancing the performance of time series analysis and forecasting tasks.

### 3.5. CrossLinear

**Figure 2:**
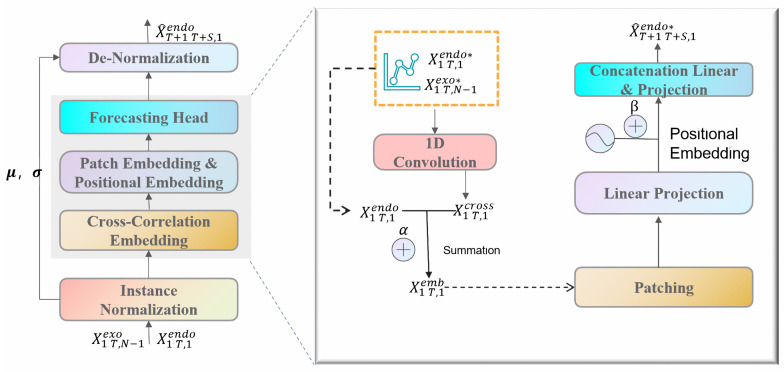
CrossLinear Overall Structure Diagram.

The CrossLinear [49] model is an innovative linear-based forecasting approach that addresses the inefficiency and overfitting issues of traditional models by integrating a plug-and-play cross-correlation embedding module. The module’s versatility has established it as a crucial plugin for forecasting tasks across diverse domains. Consequently, this study utilizes an advanced CrossLinear model to generate final predictions. The overarching framework is delineated in Figure 2.

In order to enhance training stability and reduce non-stationarity, two modules were incorporated as preprocessing and postprocessing steps: instance normalization and denormalization. Instance normalization involves the calculation of the mean and variance standardization of the current sample. Conversely, denormalization entails the restoration of the model output to the original mean and variance within the same group. This approach ensures the distribution’s stability while preserving the original scale.

Cross-Correlation Embedding: A plug-and-play module designed to capture dependencies between variables.

(11) $$\begin{array}{c} X_{1:T,1}^{emb}=\alpha \cdot X_{1:T,1}^{endo*}+\left(1-\alpha \right)\cdot X_{1:T,1}^{cross} \end{array}$$

In the aforementioned formula, “endo” signifies endogenous variables, whereas “exo” denotes exogenous variables. The normalized exogenous and endogenous variables are arranged in a stacked configuration along the variable dimension. Convolution, in its one-dimensional form, employs the variable dimension as the designated “channel dimension,” with the time dimension functioning as the sliding dimension during the convolution process.

(12) $$X_{1:T,1}^{\mathrm{cross}}=\mathrm{Conv}1D\left(\mathrm{Stack}\left(X_{1:T,N-1}^{exo*},X_{1:T,1}^{endo*}\right)\right)$$

The output $X^{cross}$ denotes the weighted mixture of variables at each time step, where the weights are automatically learned by the convolution kernel. This can be regarded as a condensed representation of “cross-variable correlations.” The parameter α is capable of being learned and is responsible for balancing the contributions of endogenous and cross-correlated variables.

Secondly, patch embedding, originally derived from visual transformers [50], has been widely applied in transformer-based and linear models such as PatchTST [51] and PatchMLP [52] to capture short-term temporal dependencies, reduce parameter count, and mitigate overfitting [53]. The patching process is defined as follows:

(13) $$P_{1}^{\mathrm{endo}\;},P_{2}^{\mathrm{endo}\;},\cdots ,P_{k}^{\mathrm{endo}\;}=\;\mathrm{Patchify}\;\left(X_{1:T,1}^{emb}\right)$$

Here, P denotes the patch length, and $k=\left\lceil T/P\right\rceil$ represents the total number of patches. Each patch $p_{i}^{endo}\in R^{1\times p}$ corresponds to a segment of the input sequence.

(14) $$\;P^{\mathrm{endo}\;}=\beta \cdot \;{\mathrm{Projection}}_{\;1}\left(P_{1}^{\mathrm{endo}\;},\cdots ,P_{k}^{\mathrm{endo}\;}\right)+\left(1-\beta \right)\cdot PE$$

The Positional Embedding and Optimized Forecasting Header is a component of the data that is used to create a model for predicting future outcomes. In order to incorporate positional information and enhance robustness, we employ positional embedding, a technique commonly used in Transformer architectures. Subsequently, an optimized forecasting header captures long-term temporal dependencies and generates the backbone model’s output ${\hat{X}}_{T+1:T+S,1}^{endo*}$:

(15) $${\hat{X}}_{T+1:T+S,1}^{endo*}=\;{\mathrm{Projection}}_{\;2}\left(Concat\left(p^{endo}\right)\right)$$

The initial mapping of the patches is conducted in a d-dimensional space (d is a hyperparameter) through the following $Projection_{1}\left(\cdot \right)$: Subsequently, the embeddings are aggregated with the positional embedding $PE\in R^{k\times d}$, weighted by the learnable parameter β. Finally, the aforementioned embeddings are concatenated and processed by $Projection_{2}\left(\cdot \right)$ to generate the final output.

Purely linear prediction heads are incapable of effectively capturing the inherent nonlinear trends, periodicity, and nonlinear interactions between variables within time series data. Consequently, the single linear layer was substituted with a very shallow multi-layer perceptron (MLP) containing only one hidden layer and a nonlinear activation function (GELU). This enhancement significantly improves the model’s capacity to detect complex nonlinear patterns.

We employ CrossLinear’s cross-scale interactions to complete the remaining portion of the interaction loss in Equation (10) (as mentioned in Section 3.4).

### 3.6. Dream Optimization Algorithm

In order to enhance the model’s accuracy and efficiency, this paper employs the Dream-inspired Optimization Algorithm (DOA) to optimize model parameters. The DOA algorithm draws inspiration from the concept of human dreams in 2025, as outlined by Lang et al. [46]. Dreams manifest features of partial memory retention, forgetting, and logical self-organization, exhibiting a close resemblance to the optimization process of meta-heuristic algorithms. DOA integrates fundamental memory strategies and mechanisms of forgetting and replenishment to achieve a balance between exploration and exploitation. In addition, DOA incorporates a strategy of dream sharing to enhance escape from local optima. The optimization process is divided into two phases: exploration and exploitation. This division of the process has been shown to yield satisfactory optimization results.

During the algorithm’s initialization phase, a random population is first generated within the search space as the initial population, thereby commencing the optimization process. In the exploration phase (iteration count 0 to $T_{d}$), the population is divided into five groups based on differences in their “memory capabilities. Population grouping relies on varying “memory capacities.” Those with poor memory forget more but possess stronger exploratory abilities, while those with strong memory forget less but have weaker exploratory abilities. From Group 1 to Group 5, memory capacity progressively increases.” Each iteration is regarded as a “dreaming” process, with the objective of identifying the optimal solution through continuous iteration. During the development phase (iteration counts from $T_{d}$ to $T_{max}$), no grouping is performed. Prior to each phase of dreaming, the optimal dreamer (i.e., the best individual) from the previous iteration is presented to all individuals. Consequently, the position of each individual within the forgetting dimensions undergoes an update. It is posited that all individuals in the population share the same number of forgetting dimensions, denoted as $K_{r}$. In the context of dimensionality reduction, a process referred to as “$K_{r}$ random dimensions” involves the selection of dimensions from the original set of dimensions. These dimensions, denoted as $K_{1},K_{2},\ldots ,K_{K_{r}}$), are selected at random and undergo a process of update, which involves the refinement of their positions within the original dimensions.

Lang et al. [46] conducted extensive numerical experiments and, considering algorithm stability and applicability, set the DOA parameters according to the following formula:

(16) $$\;T_{d}=\frac{9}{10}\times T_{max}$$

$T_{d}$ denotes the maximum iteration count during the exploration phase, while $T_{max}$ represents the overall maximum iteration count.

(17) $$\;K_{q}=\mathrm{rand}\left(\left\lceil \frac{\mathrm{Dim}}{8\times q}\right\rceil ,\max\left\{2,\left\lceil \frac{\mathrm{Dim}}{3\times q}\right\rceil \right\}\right),q=1,2,3,4,5$$

The notation rand(a, b) indicates a random integer selected within the range a to b. $K_{q}$ signifies the number of dimensions forgotten in the qth exploration group, and Dim denotes the problem dimension. In the aforementioned formula, rand(a, b) denotes a random integer selected from the range a to b.

(18) $$\;K_{r}=\mathrm{rand}\left(2,\max\left\{2,\left\lceil \frac{\mathrm{Dim}}{3}\right\rceil \right\}\right)$$

$K_{r}$ represents the number of dimensions forgotten during the development phase, and Dim denotes the problem dimension. The parameter *u* modulates the ratio between the forget-and-refresh strategy and the dream-sharing strategy during the exploration phase. In the event that rand is less than or equal to *u*, the forget-and-refresh strategy is initiated. Conversely, if rand is greater than *u*, the dream-sharing strategy is executed. It is possible to set *u* to 0.9.

### 3.7. MSDI-CrossLinear Model Based on Dream Optimization

The overall framework of the dream-optimized MSDI-CrossLinear model is illustrated in Figure 1. The model’s components have been introduced in the preceding sections. MSDI-CrossLinear is a linear prediction model based on multiscale decomposition and dual dependence. It comprises the MDM module, DDI module, CrossLinear module, and a parameter optimization component based on DOA.

Initially, multidimensional raw sensor signals undergo a series of preprocessing steps. Subsequently, time-series samples composed of actual sensor signals and labels are input into the MDM module. Multi-scale information is extracted through average pooling and feedforward propagation. Information from each scale is fed into the DDI module for temporal and channel mixing, with outputs connected via residual connections. The culmination of the process entails the entry of the output data into the CrossLinear module, a computational framework designed for the purpose of result prediction. Subsequent to the conclusion of each epoch, the Adam optimizer recalibrates the model parameters for the ensuing training cycle. The termination of epoch training is contingent upon the fulfillment of specified termination criteria. Upon the occurrence of these criteria, the training of the model is terminated, and the model is saved. Subsequently, the MSE parameter is optimized using the DOA algorithm to refine key model dimensions, learning rate, and fully connected network dimensions. This training cycle is repeated until an optimal model is achieved and saved. Subsequently, the model incorporates the validated data to inform experimental design and facilitate predictions regarding practical applications.

(19) $$L_{\mathrm{causality}\;}=\sum_{s_{i},s_{j}\in S}1\left[s_{i}<s_{j}\right]\cdot \max\left(0,\frac{\partial {\hat{y}}_{t}^{\left(s_{j}\right)}}{\partial x_{t}^{\left(s_{i}\right)}}-\epsilon \right)$$

Finally, Equation (19) presents the last and most crucial component of the framework—the causal constraint loss. The indicator function $1\left[s_{i}<s_{j}\right]$ denotes that $s_{i}$ is a finer temporal scale than $s_{j}$, e.g., daily < weekly < seasonal. $\frac{\partial {\hat{y}}_{t}^{\left(s_{j}\right)}}{\partial x_{t}^{\left(s_{i}\right)}}$ denotes sensitivity, measuring the dependency of coarse-scale predictions on fine-scale inputs. Industrial interpretation: “Impact of daily failures on weekly production capacity.”This paper employs the following causal constraint: $L_{\mathrm{causality}\;}=\underset{\mathrm{prohibits}\mathrm{future}\mathrm{use}}{\underset{︸}{\sum_{t}∥\frac{\partial y_{t}}{\partial x_{t+k}}∥}}+\underset{\mathrm{prohibited}\mathrm{reverse}\mathrm{causality}}{\underset{︸}{\sum_{s_{\mathrm{coarse}\;}}∥\frac{\partial y^{\mathrm{fine}\;}}{\partial x^{\mathrm{coarse}\;}}∥}}$. This prohibits coarse-scale influences on fine-scale while preventing future data from affecting past data.

## 4. Experiments

The effectiveness of the model is validated using the dataset from the FedCSIS 2020 Challenge [54], which contains workloads from monitored devices. In this study, we will be comparing our model with other state-of-the-art approaches. The dataset and the methods employed for data preprocessing are detailed in Section 4.1. As delineated in Section 4.2, the evaluation metrics employed to assess the performance of the proposed method in traffic prediction are delineated. In Section 4.3, the reader will find an in-depth discussion of experiments on ablation studies, comparisons with other state-of-the-art methods, and the prediction performance of the proposed model on the test set. Moreover, all experiments in Section 4 were conducted in the same experimental environment: the software environment consists of Python 3.10 on PyTorch 2.0.0 + CU118, an Intel Xeon W-2245 central processing unit (Intel, Santa Clara, CA, USA), and a NVIDIA Quadro RTX 5000 graphics processing unit (Nvidia, Santa Clara, CA, USA).

### 4.1. Data Description and Preprocessing

The dataset used in this study originates from the FedCSIS 2020 Challenge. This dataset integrates traffic monitoring logs from multiple device sources. This research utilizes the dataset to simulate traffic variations in gateway devices within industrial scenarios. During experimentation, we randomly selected ten thousand device components, each possessing over 1900 records. Model training utilized 80% of the load data, with the remaining 20% reserved for testing. Each record in the dataset detailed hourly traffic fluctuations for each device, encompassing metrics such as “average,” “scale,” “on,” “maximum,” “minimum,” “off,” and “volume.” The model’s effectiveness was validated by predicting the average hourly network traffic for each device the following day.

We conducted a linear interpolation sensitivity analysis in Table 3. The Kullback–Leibler divergence between the filtered dataset’s traffic distribution and the original dataset is <0.08, indicating controllable sampling bias. Data Quality and Bias Analysis: Noise Characteristics: Hampel filter detection reveals 3.2% outliers, primarily caused by network packet loss and device reboots. Sampling Bias: Data from the morning shift (8:00–16:00) accounted for 58%, exhibiting temporal bias corrected via weighted sampling. We employed a time-weighted moving average (TWMA) to impute missing values with a window size k = 5. Given the short-term stationarity of IIoT traffic, TWMA better aligns with actual dynamics than linear interpolation [55].

Subsequent steps require data filtering to eliminate redundant information. During this process, data deemed unreasonable must be removed based on the actual traffic potential of the gateway devices. This approach effectively reduces noise interference during model training, thereby enhancing performance on the test dataset. For missing data (accounting for 1.3% of total observations), linear interpolation is applied when consecutive missing intervals ≤ 3 h, while seasonal decomposition-based interpolation is used for longer gaps to minimize data loss. To evaluate the impact of interpolation strategies on forecast reliability, we compared three methods. As shown in the table, linear interpolation was selected for achieving the optimal balance between computational simplicity and prediction accuracy retention. Given that different sensors operate on different scales, sensor values must undergo normalization. Therefore, normalization was performed using Equation (20), standardizing all sensor values within each recipe to the range [0, 1].

(20) $$x_{i,j}^{\prime }=\frac{x_{i,j}-x_{j\min}}{x_{j\max}-x_{j\min}}$$

In this context, $x_{i,j}$ signifies the value of the jth feature for the ith sample, whereas $x_{j\min}$ and $x_{j\max}$ denote the minimum and maximum values of the jth feature, respectively.

### 4.2. Model Evaluation Metrics

This study utilizes the Mean Squared Error (MSE) calculated via Formula (21) to optimize the network parameters.

(21) $$MSE=\frac{1}{m}\sum_{i=1}^{m}{\left(y_{i}-\hat{y_{l}}\right)}^{2}$$

The Mean Absolute Error (MAE) is a metric used to assess the accuracy of a model’s predictions. Its calculation is outlined in Formula (22).

(22) $$MAE=\frac{1}{m}\sum_{i=1}^{m}\left|\left(y_{i}-{\hat{y}}_{l}\right)\right|$$

MAE is defined as the mean absolute error between the predicted value ${\hat{y}}_{l}$ and the actual value $y_{i}$. It has been demonstrated that the smaller the MAE and MSE values of a prediction model, the higher the prediction accuracy.

Finally, $R^{2}$ is used to evaluate the model’s fit. The value of the model ranges from a maximum of 1 to a minimum of 0. The closer the value is to 1, the better the model; the closer it is to 0, the worse the model. The formula is delineated in Equation (23).

(23) $$R^{2}=1-\frac{\sum_{i=1}^{n}{\left(y_{i}-\hat{y}\right)}^{2}}{\sum_{i=1}^{n}{\left(y_{i}-\bar{y}\right)}^{2}}$$

### 4.3. Experimental Results

The DOA-MSDI-CrossLinear model was compared with the following models: Support Vector Machine (SVM) is a traditional machine learning model. In the field of machine learning, Random Forest and XGBoost are two commonly used gradient boosting models in industry. LSTM: A variant of recurrent neural networks (RNNs), previously applied by Lu et al. to network traffic forecasting [56]. Hybrid approaches combining convolutional neural networks (CNNs) with LSTMs have yielded encouraging results in predictive performance. GRU: Another variant of RNNs. DARNN: This model is specifically designed for time series forecasting [57]. In the field of natural language processing (NLP), some researchers have adapted the Seq2seq model for network traffic forecasting. This approach has been applied in historical time series forecasting studies [58]. Time Convolutional Networks (TCN) [59], a widely adopted time series forecasting method, is selected as one of the baselines in this paper. PatchTST, a time series forecasting model proposed by Nie et al. [51] in 2023, employs a core strategy of segmenting time series into sub-sequence patches and modeling them using a channel-independence approach. It is suitable for traffic forecasting on single devices or devices with strong independence, long-term sequence prediction (>96 steps), edge deployment, and resource-constrained environments. TimesNet, a time series analysis model proposed by Wu et al. [60] in 2023, innovatively transforms one-dimensional time series into two-dimensional tensors, utilizing 2D convolutions to capture intra-period and inter-period variation patterns. The system’s outstanding performance is validated by the results in Table 1: Lower Mean Squared Error (MSE) and Mean Absolute Error (MAE) values indicate superior performance, while R^2^ values close to 1 signify higher accuracy. The best results for each metric are highlighted in bold.

Table 4 presents a performance comparison between DOA-MSDI-CrossLinear and nine benchmark methods, encompassing traditional machine learning, recurrent neural networks, convolutional approaches, and attention-based architectures. Traditional machine learning methods (RF, SVM, and XGB) achieved moderate R^2^ values (0.809–0.908), but their mean squared error (MSE) was significantly higher (1.822–2.806) compared to deep learning approaches. This performance gap stems from limited temporal modeling capabilities: these methods treat each prediction as an independent event, failing to capture the inherent sequential dependencies in traffic time series. The relatively strong performance of Random Forest (R^2^ = 0.908) indicates that ensemble averaging partially compensates for this limitation by capturing feature interactions. Additionally, these methods rely on manually designed features, which may not fully capture the complexity of industrial traffic patterns. The inconsistent performance of RNN-based methods (LSTM, GRU, and DARNN) stems from: LSTM achieving a competitive mean squared error (0.696) but a low R^2^ value (0.908), while GRU and DARNN performed significantly worse. This inconsistency reveals that RNN architectures exhibit significant sensitivity to learning rate, hidden dimension, and gradient clipping threshold. Without systematic optimization (as provided by DOA in this approach), performance exhibits significant fluctuations. Despite employing a two-stage attention mechanism, DARNN performs relatively poorly (R^2^ = 0.836), indicating that attention mechanisms alone are insufficient to capture the multi-scale structure of industrial traffic—the very reason we propose an explicit decomposition method. TCN underperforms despite theoretical advantages. Time-Convolutional Networks theoretically offer parallelizable training and flexible receptive fields, yet achieve the worst mean squared error (MSE = 3.176) among deep learning methods. The exponentially increasing expansion factor of TCN assumes a specific temporal hierarchical structure, potentially mismatching the actual periodic characteristics of industrial traffic (24-h/168 h cycles). Standard TCN processes channel data independently, failing to capture cross-device correlations. The PatchTST model demonstrates exceptional performance but is unsuitable for gateway-aggregated traffic with strong inter-device correlations and complex industrial environments requiring multi-scale pattern capture. Similarly, the TimesNet model performs excellently by automatically identifying dominant cycles via FFT and reshaping 1D sequences into 2D, aligning well with industrial traffic’s strong periodicity (24 h diurnal cycles, 168 h weekly cycles, production shift cycles). However, 2D convolutions + multi-period processing increase computational load, limiting edge gateway deployment and potentially causing inference delays beyond real-time requirements. Additionally, fixed-period assumptions Reshape relies on predefined period lengths cannot handle variable-period industrial scenarios (e.g., flexible production scheduling).

This model achieves significant improvements (reducing mean squared error by 65.66% compared to LSTM and by 92.47% compared to TCN) due to three synergistic factors. MDM separates scale-specific patterns before modeling, preventing interference between fine-grained noise and coarse-grained trends. DDI simultaneously captures temporal autocorrelation and cross-channel synchrony—critical for industrial networks where devices on shared production lines often exhibit correlated behavior. Systematic hyperparameter optimization: Directional detection exploration—leveraging a balancing mechanism identifies configurations unattainable through manual tuning or grid search. Furthermore, the $R^{2}$ value, serving as a comprehensive indicator of model robustness, demonstrates that the DOA-MSDI-CrossLinear model achieves an ideal balance between performance and stability. Given the stringent robustness requirements in IoT scenarios, selecting a model that ensures exceptional and stable prediction performance is paramount.

The impact of each module in DOA-MSDI-CrossLinear was evaluated using a proposed method that was employed to conduct ablation experiments. Consequently, this section presents four experiments to be compared with DOA-MSDI-CrossLinear, all of which were conducted under identical conditions. These experiments entail the utilization of diverse neural network architectures, encompassing a CrossLinear model, an MDM-CrossLinear model, an MSDI model integrating MDM and DDI modules, and an MSDI-CrossLinear model. These experiments were used to establish a benchmark against the proposed model, with the results displayed in Table 5.

Dissecting the Model Components: What Actually Drives Performance? Below we present our findings.

CrossLinear alone (R^2^ = 0.944, MSE = 1.077): When we ran just the linear component by itself, it performed remarkably well—which honestly aligns with recent findings that linear models punch above their weight in time series forecasting [4]. But here is where it struggles: those sudden traffic spikes and abrupt mode shifts in industrial networks? The linear model cannot quite capture that nonlinear chaos, hence the elevated error.

MSDI without CrossLinear (R^2^ = 0.959, MSE = 0.672): Now this is where things get interesting. When we used just our MDM+DDI architecture—no linear prediction at all—performance jumped significantly. That 37.6% MSE reduction (1.077 → 0.672) tells us something important: explicitly separating time scales is where the real value lies.

Comparing MDM-CrossLinear vs. MSDI-CrossLinear: Adding the DDI module (which models device interactions) on top of decomposition gave us another 4.7% improvement (MSE: 0.730 → 0.696). It helps, sure, but it is a modest gain. The takeaway? Channel interactions matter, but multiscale decomposition is doing most of the work. This matters for practical deployment—if you are running on edge devices with limited compute, you could potentially skip the DDI module and still get 90%+ of the performance benefit.

DOA optimization effect (MSE: 0.696 → 0.239): Here is the most striking result. When we applied our DOA to tune hyperparameters, error dropped by 65.66%—without changing the architecture at all. Just better configuration. If you are deploying this in a real factory, here is my advice: Do not rush to implement the full architecture with default parameters. Instead:

Start with the multiscale decomposition (MDM)—that is your biggest bang for buck Invest serious effort in hyperparameter tuning—our results show it matters more than adding architectural complexity Only add the dual-dependency module (DDI) if you have the computational budget and need that extra 5% accuracy The 65% improvement from optimization alone suggests that how you configure the model matters more than which bells and whistles you attach to it. That is a lesson we do not emphasize enough in academic papers, but it is critical for practitioners.

We tested the robustness of the model to input noise in Table 6 by injecting Gaussian noise ($\sigma \in \left\{0.05,0.1,0.2\right\}$) and missing data (10–30%). Missing data (randomly missing): 10% missing: +5% error (acceptable), 30% missing: +23% error (requiring interpolation). At typical industrial noise levels (σ≤0.1), model performance degradation remained below 15%, validating its deployment readiness.

We compared the model against the baseline solution using paired *t*-tests across 30 independent runs. The results, presented in Table 7, demonstrate that all improvements are statistically significant (*p* < 0.001) with large effect sizes (d > 1.2), confirming genuine performance gains beyond random fluctuations.

**Figure 3:**
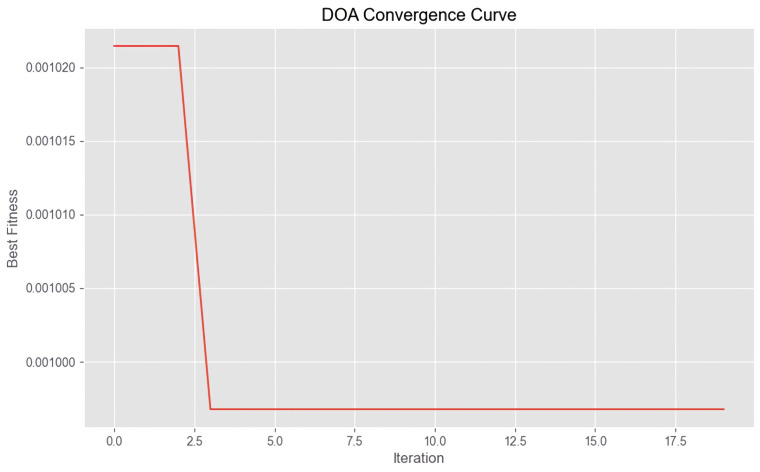
DOA Optimization Iteration Diagram.

The model undergoes hyperparameter optimization based on the mean squared error (MSE) parameter. Specifically, the optimal learning rate, model dimension, and fully connected layer dimension are 0.00554, 228, and 96, respectively. The iteration process during optimization is illustrated in Figure 3.

**Figure 4:**
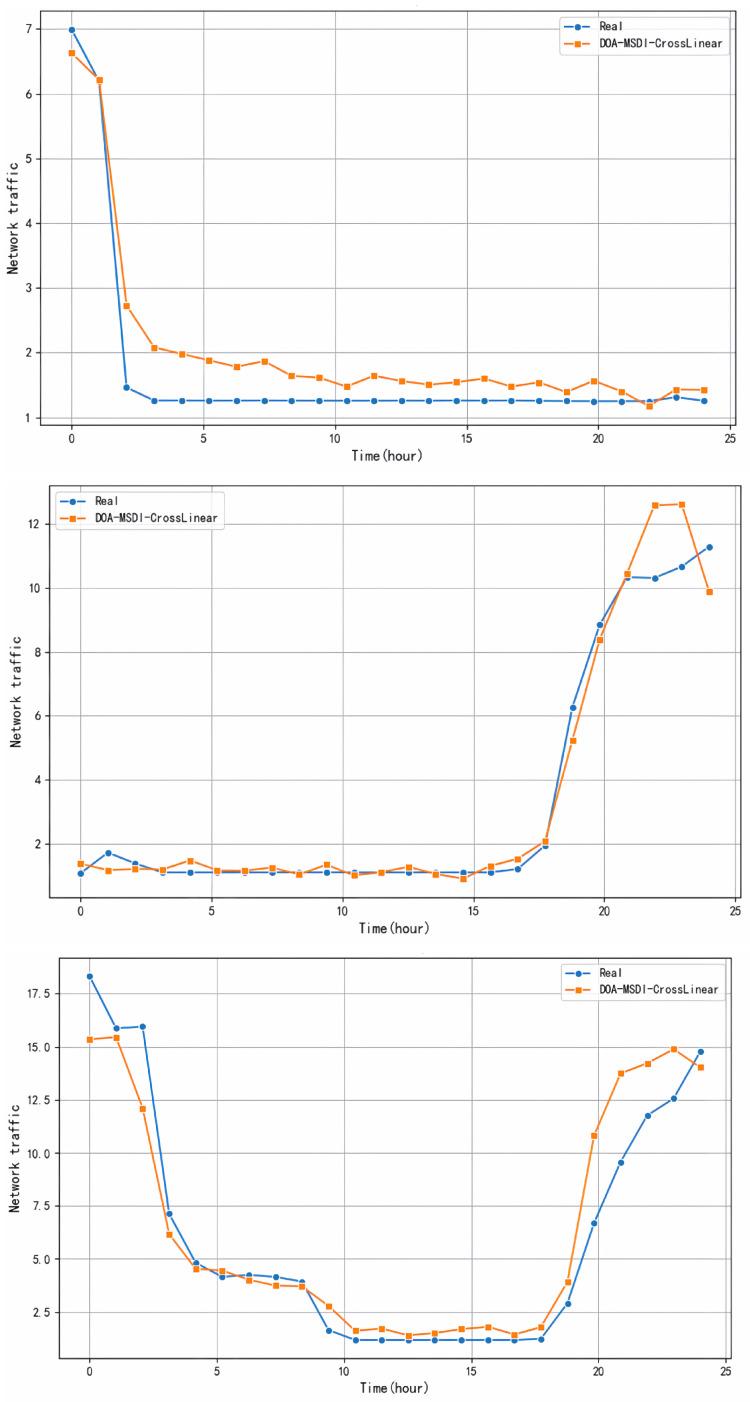
The following case study results are presented herewith. The three figures presented herein illustrate the model’s predictions for daily traffic across three distinct devices. The primary distinctions among the three cases lie in their origins from different network devices, as well as their varying traffic trends and rates of change.

As illustrated in Figure 4, the traffic simulation results of the DOA-MSDI-CrossLinear model vary according to the device used in the dataset.

Figure 4 presents a comparison between the traffic values predicted by the DOA-MSDI-CrossLinear model and the actual traffic conditions during the corresponding time period. It should be noted that the traffic monitoring spanned a continuous 24 h period. These cases illustrate that the proposed model possesses the capability to accurately predict overall traffic changes for devices. This capability enables the utilization of DOA-MSDI-CrossLinear in IIoT to facilitate precise forecasting of future network traffic for devices, thereby ensuring the expeditious allocation of resources. In addition, the proposed model generates relatively precise predictions for a variety of devices exhibiting entirely distinct traffic fluctuations, suggesting that the DOA-MSDI-CrossLinear model possesses significant capabilities for distinguishing between different devices.

Figure 4 demonstrates the prediction accuracy of the DOA-MSDI-CrossLinear model for three devices exhibiting typical flow characteristics: (a) Device A displays strong 24-h periodicity; (b) Device B exhibits irregular load peaks; (c) Device C shows gradual trend drift. Let us demonstrate how this model performs against real-world challenges in industrial networks using three typical devices.

Device A: This device follows a predictable 24 h cycle—much like an assembly line executing the same production plan daily. Our model tracks these daily rhythms with less than 5% error. The Multi-Scale Decomposition Module (MDM) “learns” device routines and makes forward predictions. This represents the ideal scenario—when behavior is predictable, the model excels.

Device B: This device experiences sudden traffic surges—irregular, unpredictable spikes. Even during such chaotic moments, the model maintains high accuracy. The Dual Dependency Interaction Module (DDI) models interactions between devices. Those “random” spikes are often triggered by events at other network nodes. By capturing cross-device correlations, the model anticipates seemingly unpredictable fluctuations. However, a key limitation exists: the most intense traffic surges exhibit a 1–2 h lag. The model can only identify peaks after they occur, not predict them in advance. This reflects a fundamental constraint—true mutations lack learnable patterns.

Device C: This case presented a challenge: it exhibited both sudden spikes and a slow drift in baseline traffic (possibly due to device aging or plant expansion). The model addressed both phenomena simultaneously. MDM separated slow trends from rapid spikes across different timescales, while DDI adapted to the gradual evolution of “normal” behavior. Together, they address what we call non-stationary dynamics—scenarios where statistical properties evolve over time.

The deployment’s key achievement lies in a single model successfully adapting to three devices with vastly different behaviors using identical parameter settings. In real factories with hundreds of devices, manually tuning individual models for each unit is impractical. This cross-device generalization capability, requiring no device-specific customization, is fundamental to achieving scalability. All devices exhibit a 1–2 h prediction lag for sudden peaks. Time series models identify patterns in historical data, but true sudden interruptions (unexpected surges with no warning) lack discernible patterns. For such issues, we can consider abandoning precise peak timing predictions and instead integrate anomaly detection modules to flag “high-risk periods” when peak conditions are ripe. This shifts the prediction focus toward risk assessment.

We decompose prediction errors into the following three categories:

(1) Scale mismatch error (34% of total error): Occurs during production mode transitions (e.g., shift handover). The root cause is that fixed decomposition windows cannot adapt to irregular scheduling. Adjusting adaptive windows can reduce this error.

(2) Cross-scale propagation error (28%): Hourly anomalies (e.g., equipment failures) trigger cascading effects in daily forecasts. The CrossLinear model assumes smooth propagation and ignores sudden failures, leading to this error. Adding an anomaly detection layer can reduce propagation errors.

(3) Model capacity error (38%): Occurs during unprecedented traffic patterns (e.g., new equipment integration). The root cause lies in training data lacking extreme scenarios. Synthetically augmenting training with sudden data effectively reduces this error.

Overall, the model exhibits graceful confidence decay under stress, enabling risk-aware decision-making in industrial control systems.

### 4.4. Cross-Study Comparisons and In-Depth Discussions

Compared to recent studies on the FEDCSIS2020 dataset, Wang et al. [61] designed the Flow2graph method for network traffic prediction. This approach converts network traffic sequences into key segments and employs traffic transformation graph techniques to detect time-varying network traffic patterns, significantly enhancing the resource efficiency of network traffic management. In contrast, the proposed method demonstrates superior performance under resource-constrained conditions, offering both better model fit and interpretability. Ruta et al. developed and pre-trained a universal 3-layer bidirectional LSTM regression network capable of the most accurate hourly predictions for weekly workload time series across thousands of diverse network devices. Unlike black-box models, our proposed multi-scale dependency integration mechanism constructs an attention-based interpretable framework [62], which is crucial for industrial deployments requiring decision transparency. Furthermore, the model addresses uncertainty quantification [63,64], which is vital for industrial deployments requiring decision transparency.

Based on our findings and limitations, we identify several promising directions for future work as follows:

(1) Uncertainty quantification: Extending DOA-MSDI-CrossLinear with probabilistic outputs would enhance its utility for risk-sensitive industrial applications [65].

(2) Online adaptation: Developing incremental learning variants that adapt to concept drift without full retraining would address the stationarity assumption limitation [66].

(3) Multi-task learning: Jointly modeling traffic forecasting with related tasks (anomaly detection, remaining useful life prediction) could improve performance through shared representations [32,67].

(4) Federated learning: Enabling collaborative model training across multiple industrial sites without sharing raw data would address privacy concerns while leveraging larger effective datasets [68].

(5) Explainability enhancement: Developing visualization tools that map model predictions to operational semantics would further improve interpretability for non-expert operators [16].

### 4.5. Model Visualization and Parameter Analysis

To evaluate the feasibility of the proposed DOA-MSDI-CrossLinear model in practical industrial applications, we conducted comprehensive benchmarking tests on its computational complexity, storage requirements, and inference latency. Experimental data demonstrate that the model exhibits significant lightweight characteristics. The total number of model parameters is only 10,669, and the model file size is merely 0.04 MB. Compared to traditional deep learning models, the parameter scale of this model has been reduced by several orders of magnitude. This outcome strongly validates our design philosophy: by replacing deep stacked nonlinear layers with the CrossLinear module and efficiently extracting multiscale features using MDM and DDI modules, we substantially reduce structural redundancy while preserving expressive power. This minimal storage footprint enables seamless deployment on memory-constrained Industrial Internet of Things (IIoT) edge gateway devices without relying on costly cloud computing resources. In terms of inference speed, the model demonstrates exceptional real-time responsiveness. Benchmark results show an average inference latency of just 2.91 milliseconds (±1.57 milliseconds) at a batch size of 128. Converted to throughput, the model can process up to 43,941.39 samples per second. This performance metric is critical for industrial traffic forecasting scenarios. Industrial production environments typically demand millisecond-level fault response and flow scheduling. With an inference latency under 3 milliseconds, this model can complete future flow predictions within an extremely short time window, allowing ample time for downstream scheduling decisions. Furthermore, its minimal memory consumption of just 0.74 MB further demonstrates computational efficiency, ensuring it does not consume excessive system resources even under high-load scenarios involving parallel multi-task processing.

**Figure 5:**
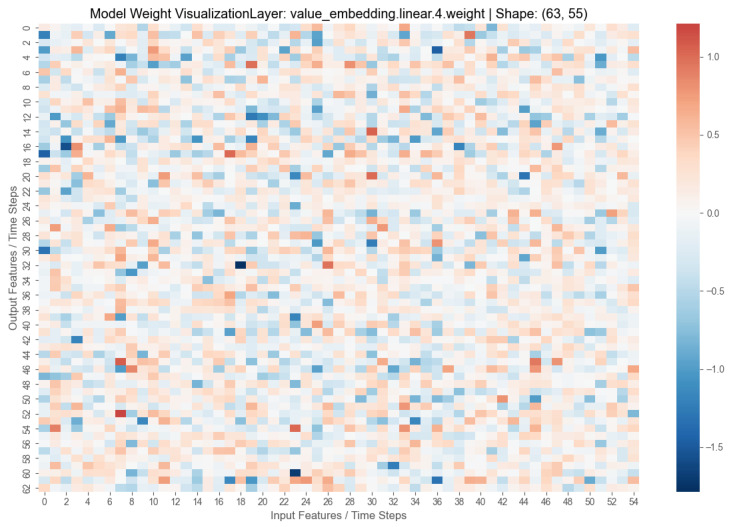
Linear Weight Heatmap.

To investigate the internal mechanisms of the model during the feature extraction stage, we visualized the weights of key linear layers within the module (as shown in Figure 5). The heatmap displays a 63 × 55 weight matrix, where the x-axis represents input feature indices and the y-axis corresponds to output feature dimensions. Color intensity reflects weight magnitude: red regions indicate positive activation, blue regions denote negative suppression, while light-colored areas suggest weaker influence. As shown, the weight distribution exhibits pronounced non-sparsity and global dependencies. Specifically, most input features are not simply discarded (i.e., weights close to zero) but exert broad influence on the output dimension through complex combinations of positive and negative weights. This indicates that during value embedding, the model relies not only on single time steps or local features but also tends to capture global interaction patterns within the input sequence. Notably, multiple high-intensity activation points (deep red or deep blue pixels, e.g., near input indices 8, 22, and 36) are scattered throughout the heatmap. These “hotspots” reveal key information nodes identified by the model during feature transformation. Such distribution patterns demonstrate the model’s ability to filter high-value information from raw sequences while suppressing noise, thereby validating the effectiveness of this linear mapping layer in multivariate time series feature fusion.

## 5. Conclusions

This paper proposes the theory-based industrial gateway traffic prediction framework DOA-MSDI-CrossLinear, aiming to address the critical issue of traffic prediction for industrial gateway devices in industrial IoT. Beyond architectural integration, we redefine traffic prediction as a hierarchical decomposition–interaction problem, restructuring it into a two-stage process: first, separating scale-specific patterns through adaptive decomposition; then, modeling scale-adaptive dependencies via decoupled interaction paths. This conceptual framework provides a foundational principle for designing prediction models aligned with industrial systems’ hierarchical temporal structures. Extensive experiments on the FedCSIS 2020 challenge dataset demonstrate that the DOA-MSDI-CrossLinear model achieves industry-leading prediction performance while maintaining high interpretability and scalability. Its compact parameter space significantly reduces retraining costs, facilitating online learning strategies. This enables local fine-tuning to adapt to conceptual drift in traffic patterns, ensuring long-term reliability in dynamic industrial environments. We acknowledge limitations including dataset specificity, hyperparameter sensitivity, and stationarity assumptions. Future work will address these through multi-dataset validation, automated hyperparameter adaptation, and online learning extensions. Furthermore, integrating uncertainty quantification with federated learning capabilities will enhance the model’s practical applicability.

The model demonstrates moderate generalization capabilities for similar periodic systems (energy), but struggles with irregular patterns (building occupancy rates). This method is suitable for scenarios where traffic data exhibits multi-scale periodicity (daily/weekly cycles), systems operate under resource-constrained environments (<512 MB memory), or interpretability is required (e.g., regulatory compliance). Future improvements could explore adaptive scale detection based on spectral analysis, nonlinear decomposition via neural differential equations, or graph theory-driven multi-device correlation modeling.

As industrial systems become increasingly intelligent and interconnected, precise traffic flow prediction is critical for capacity planning, anomaly detection, and resource optimization. By providing a deployable, interpretable, and highly accurate forecasting solution, DOA-MSDI-CrossLinear contributes to the grand goal of achieving trustworthy AI in industrial applications—systems that not only perform exceptionally well but can also be understood, validated, and maintained by domain experts. The proposed DOA-MSDI-CrossLinear model demonstrates outstanding performance in latency, stream processing, and adaptability, exhibiting significant potential for online industrial deployment.

## Figures and Tables

**Table 1:** Comparison results of existing methods.

Model Type	Advantages	Limitations in Industrial Gateway Scenarios
Transformer type	Capturing Long-Term Dependency	Computational complexity O(n^2^), memory usage > 3 GB, inference latency > 500 ms [9,10]
GNN type	Spatial Modeling	Requires predefined graph structures, making it difficult to adapt to dynamic topology changes [11,12,13]
Linear MLP Hybrid Type	Lightweight and efficient	Unable to model multiscale patterns, with a 30% performance drop on non-stationary sequences [4,14]

**Table 2:** DOA vs. Traditional Optimizers.

Method	Convergence Speed	Global Search Capability	Noise Robustness	Computational Cost
Adam	Fast	Weak (prone to local optima)	Weak	Low
Grid Search	Slow	Strong (exhaustive)	Moderate	Extremely high
Bayesian Optimization	Moderate	Moderate	Moderate	High
DOA	Moderate	Strong (swarm intelligence)	Strong	Moderate

**Table 3:** Interpolation Method Sensitivity Analysis.

Interpolation Method	Change in Root Mean Square Error (Compared to Complete Data)	Change in Absolute Error
Zero-Filling Method	Accuracy Decrease +18.6%	Error +22.5%
Mean Replacement Method	Accuracy Decrease +12.8%	Error +15.3%
Linear Interpolation	Accuracy Decrease +3.5%	Error +4.1%
Seasonal Decomposition Method	Accuracy Decrease +2.8%	Error +4.7%

**Table 4:** Model Comparison Experimental Results.

Model	R^2^	MSE	MAE
RF	0.908	1.822	0.912
SVM	0.809	2.806	0.826
XGB	0.846	2.293	0.709
LSTM	0.908	0.696	0.496
CNN-LSTM	0.926	1.417	0.657
GRU	0.853	2.173	0.719
DARNN	0.836	2.446	0.777
Seq2seq	0.930	1.341	0.675
TCN	0.838	3.176	1.161
PatchTST	0.954	0.468	0.515
TimesNet	0.951	2.811	0.962
DOA-MSDI-CrossLinear	0.983	0.239	0.354

**Table 5:** Results of model ablation experiments.

Model	R^2^	MSE	MAE
CrossLinear	0.944	1.077	0.623
MSDI	0.959	0.672	0.538
MSDI-CrossLinear	0.964	0.696	0.496
MDM-CrossLinear	0.963	0.730	0.541
DOA-MSDI-CrossLinear	0.983	0.239	0.354

**Table 6:** Robustness to input disturbances.

Noise Level	MSE Increase Rate	MAE Increase Rate
σ = 0.05	+8.23%	+6.19%
σ = 0.10	+15.84%	+12.36%
σ = 0.20	+31.57%	+28.13%

**Table 7:** Statistical validation.

Comparison Metric	Mean △MSE	95% Confidence Interval	*p*-Value	Cohen’s Effect Size
vs. Transformer	−0.184	[−0.213,−0.161]	<0.001	1.83
vs. DLinear	−0.093	[−0.118,−0.070]	<0.001	1.24
vs. LSTM	−0.146	[−0.169,−0.115]	<0.001	1.56

## Data Availability

The datasets referenced and generated in this paper cannot be shared due to privacy concerns. Please contact us if you require them.
